# Evaluation of the Synergistic Antibacterial Effects of Fosfomycin in Combination with Selected Antibiotics against Carbapenem–Resistant *Acinetobacter baumannii*

**DOI:** 10.3390/ph14030185

**Published:** 2021-02-25

**Authors:** Ozioma F. Nwabor, Pawarisa Terbtothakun, Supayang P. Voravuthikunchai, Sarunyou Chusri

**Affiliations:** 1 Division of Infectious Diseases, Department of Internal Medicine, Faculty of Medicine, Prince of Songkla University, Hat Yai, Songkhla 90112, Thailand; nwaborozed@gmail.com (O.F.N.); pawarisa.3tk@gmail.com (P.T.); 2 Division of Biological Science, Faculty of Science and Natural Product Research Center of Excellence, Prince of Songkla University, Hat Yai, Songkhla 90112, Thailand; supayang.v@psu.ac.th

**Keywords:** *Acinetobacter baumannii*, multi-drug resistance, fosfomycin, combination therapy, antibiotic synergism

## Abstract

The spread of multi-drug resistant (MDR) pathogens and the lagging pace in the development of novel chemotherapeutic agents warrant the use of combination therapy as a reliable, cost-effective interim option. In this study, the synergistic effects of fosfomycin in combination with other antibiotics were assessed. Of the 193 isolates, 90.6% were non-susceptible to fosfomycin, with minimum inhibitory concentrations (MICs) of ≥128 µg/mL. Antibacterial evaluation of fosfomycin-resistant isolates indicated multi-drug resistance to various antibiotic classes. Combinations of fosfomycin with 12 commonly used antibiotics synergistically inhibited most fosfomycin-resistant isolates. The fractional inhibitory concentration index indicated that combining fosfomycin with either aminoglycosides, glycylcyclines, fluoroquinolones, or colistin resulted in 2- to 16-fold reduction in the MIC of fosfomycin. Time-kill kinetics further confirmed the synergistic bactericidal effects of fosfomycin in combination with either amikacin, gentamicin, tobramycin, minocycline, tigecycline, or colistin, with more than 99.9% reduction in bacterial cells. Fosfomycin-based combination therapy might serve as an alternative option for the treatment of MDR *A. baumannii*. Further steps including in vivo efficacy and toxicity in experimental models of infection are required prior to clinical applications.

## 1. Introduction

The emergence of multi-drug-resistant pathogens has limited treatment options with a consequent increase in mortality and extended hospital stays. With the rapid spread of drug resistance, especially among Gram-negative bacterial isolates, and the lag in the discovery of novel bioactive compounds, the fight against infectious diseases continues. The search for alternative chemotherapeutic agents effective for the management of multi-drug resistant (MDR) pathogens including MDR *Acinetobacter baumannii* have become an urgent public health priority, warranting prospecting for novel active compounds [1]. Attempts at revitalizing old and already existing but abandoned agents through chemical modifications and combination therapies is indicated as an interim strategy for effective management of drug-resistant pathogens [2,3]. Antimicrobial combinations have been proposed to synergistically inactivate microbial cells through several mechanisms including enhanced bioavailability, inhibitor inhibition, sequential blockade, mutual stabilization, parallel pathway inhibition, and regulation modulation [4].

*Acinetobacter baumannii*, a Gram-negative bacterium of the family Moraxellaceae, is an opportunistic pathogen frequently associated with hospital-acquired infections and disease outbreaks within hospital intensive care units. In the past, the use of carbapenems was the choice option for the treatment of infections caused by *A. baumannii.* However, recent trends in the spread of carbapenem-resistant *A. baumannii* pose a serious threat to public health and have prompted the classification of MDR *A. baumannii* as a priority and a critical antimicrobial-resistant pathogen [5]. Several antibacterial agents are indicated for the management of *A. baumannii* infections, including fosfomycin, which was recently listed as a miscellaneous agent against *A. baumannii* [6]. Limitations such as the poor penetration of colistin [7,8,9,10] and low plasma levels of tigecycline [11] discourages the use of these agents as single therapies in the management of infections caused by MDR pathogens.

Fosfomycin is an epoxide broad-spectrum antibacterial agent that inhibits cell-wall biosynthesis. Available data suggest that fosfomycin is safe and cost effective; has no clinically relevant pharmacological interactions with other agents, including drugs, stimulants, food, intravenous fluids, or peritoneal dialysis solutions [12,13]; has mild or little side effects; and has no contraindications [14]. However, since resistance to fosfomycin can be acquired in vivo when used as monotherapy, it is often administered in combination with other antimicrobial agents in systemic therapy [15,16,17]. The antibacterial potency of fosfomycin in combination with other antibiotics has been demonstrated against carbapenem-resistant *A. baumannii,* though currently available data are insufficient for substantial conclusions. A prospective clinical observational study reported an overall increase in 30-day survival in patients with severe pneumonia caused by carbapenem-resistant *A. baumannii* after treatment with fosfomycin-containing regimen [18]. Similarly, the in vivo efficacy of combination of colistin with fosfomycin in a mouse model showed synergistic effects against MDR *A. baumannii* [19]. In addition, in vitro use of fosfomycin in combination with other antibiotics suggested effective synergistic outcomes against Gram-negative bacterial isolates including *Klebsiella pneumoniae* [20,21], *Escherichia coli* [22,23], and *Pseudomonas aeruginosa* [24].

This study evaluates the effects of fosfomycin in combination with imipenem, meropenem, doripenem, colistin, amikacin, gentamicin, tobramycin, trimethoprim/sulfamethoxazole, ciprofloxacin, levofloxacin, minocycline, and tigecycline on carbapenem-resistant *A. baumannii* isolates. The potential results of this study are intended to inform health practitioners on possible antibiotic combinations to explore the effective treatment of patients with MDR *A. baumannii* infections.

## 2. Results

### 2.1. Distribution of A. baumannii Isolates

Clinical *A. baumannii* isolates were collected from patients admitted to tertiary hospitals in Southern Thailand. Most of the patients had underlying health conditions including diabetes mellitus, essential blood hypertension, dyslipidemia, chronic kidney disease, cerebrovascular disease, coronary heart disease, chronic obstructive pulmonary disease, and human immunodeficiency viral infection. Samples included sputum (129 samples), nasopharyngeal swabs (15 samples), bacteremia (14 samples), skin (14 samples), nasogastric tube (nine samples), and urine (five samples). The demographic data, clinical characteristics, and outcomes of the patients with infections due to carbapenem-resistant *A. baumannii* are presented in Table S1.

### 2.2. Antibacterial Effects of Carbapenem on Clinical Isolates of A. baumannii

**Figure 1:**
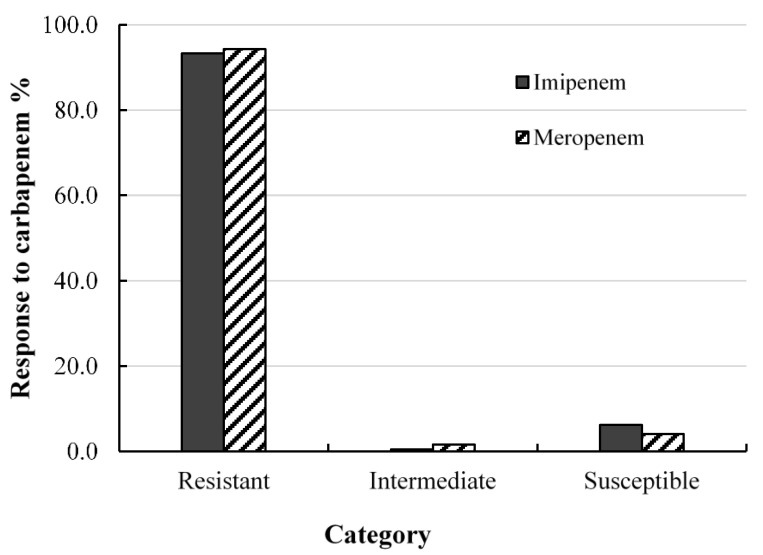
Screening for antibacterial activity of carbapenems (imipenem and meropenem) against clinical *Acinetobacter baumannii* isolates, determined using disc diffusion method.

The effects of imipenem and meropenem on the 193 clinical isolates of *A. baumannii* are presented in (Figure 1, Table S2). Based on the CLSI interpretive categories and zone diameter breakpoints (susceptible, ≥23 mm; intermediate, 20–22 mm; resistant, ≤19 mm) [25], the results revealed that 15 isolates demonstrated zones of inhibition to imipenem, while 13 isolates demonstrated zones to meropenem. Among the isolates with zones of inhibition for imipenem, SK012 was within the intermediate range, while TR013, and TR129 were resistant. On the other hand, isolates TR13 and TR129 were resistant to meropenem with zones of inhibition of ≤14 mm, while isolates SK03, TR30, and TR31 were within the intermediate range. Isolate SK04, SK23, ST06, ST23, TR01, TR51, TR96, and TR103 were susceptible to meropenem. The results showed that 12 isolates (6.2%) were susceptible to imipenem, while eight isolates (4.1%) were susceptible to meropenem.

### 2.3. Antibacterial Activities of Fosfomycin against A. baumannii Isolates

**Figure 2:**
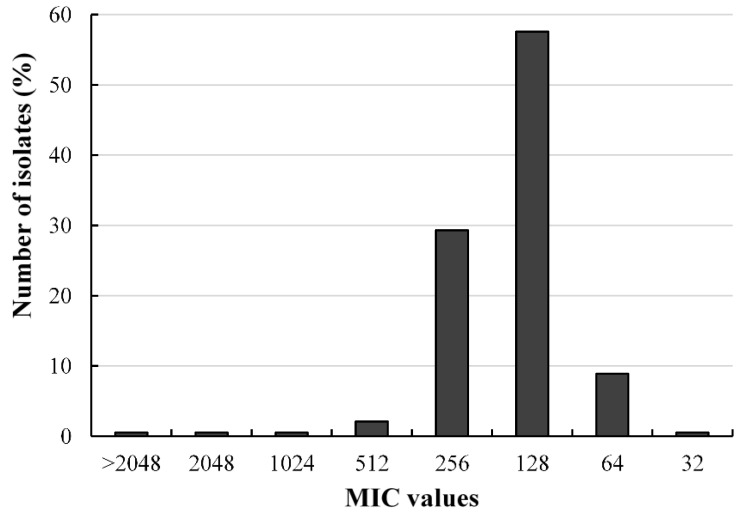
Distribution of the minimum inhibitory concentrations of fosfomycin against clinical *A. baumannii* isolates, determined by the broth microdilution technique.

The broth microdilution results of fosfomycin activity on 191 *A. baumannii* isolates are presented in Figure 2 and Table S2. The MIC_50_ and MIC_90_ were recorded at 128 and 256 µg/mL, respectively. Although fosfomycin is listed as a miscellaneous agent for the management of *A. baumannii* [6], there are currently no standard breakpoint figures for interpreting of antimicrobial activity of fosfomycin against *A. baumannii.* Several researchers have adopted CLSI breakpoint limits for *Enterobacterales* (susceptible, ≤64; intermediate, 128; resistant, ≥256) [26,27,28]. Based on the CLSI standards for *Enterobacterales*, the antibacterial effects of fosfomycin on 110 isolates were classified as intermediate, 63 isolates were resistant, while 18 isolates were susceptible. A similar high prevalence of fosfomycin resistance among *A. baumannii* isolates has been reported [26,29,30,31].

### 2.4. Antibacterial Effects of Fosfomycin-Resistant Isolates

The results of the antibacterial effects of fosfomycin against the five highly resistant isolates (MIC ≥ 512 µg/mL), evaluated by the agar dilution technique, are presented in Table 1. The results indicated agreement between the broth microdilution and agar dilution methods for all the isolates, and the standard strain ATCC 19606 was used (Table 1). Minimum inhibitory concentrations were obtained for all isolates except ST26. In addition, in the presence of the efflux pump inhibitor compound CCCP, a two- to four-fold reduction in the MIC of isolates was observed (Table 1).

### 2.5. Susceptibility Profile of Carbapenem- and Fosfomycin-Resistant A. baumannii Isolates

The antibiogram of the highly fosfomycin-resistant isolates were evaluated against 12 conventional antibiotics including carbapenems, aminoglycosides, glycylcyclines, fluoroquinolones, polymyxin (colistin), and the folate pathway antagonist (trimethoprim/sulfamethoxazole) (Table 2). The isolates showed multi-drug resistance to antibiotics across the various classes. All the isolates were highly resistant to carbapenems and trimethoprim/sulfamethoxazole but were susceptible to amikacin. The results suggested that antibiotics of the aminoglycoside class were more effective, with 60% susceptibility against the isolates. In addition, four of the isolates were resistant to colistin, a last-resort antimicrobial agent for the treatment of carbapenem-resistant *A. baumannii*, with MIC values ≥2 µg/mL. Antibiotics of the glycylcycline class also exhibited good activity against the resistant isolates. Three isolates and the standard strain ATCC 19606 were susceptible to minocycline, with MIC values ranging from 0.25 to 1 µg/mL, while four isolates were susceptible to tigecycline. Furthermore, antibiotics of the fluoroquinolone class showed limited activity against the isolates. All the isolates, including ATCC 19606, were resistant to ciprofloxacin, except isolate SK12 with an intermediate activity, whereas two isolates and the standard test strain were resistant to levofloxacin.

### 2.6. Ethidium Bromide Uptake

**Figure 3:**
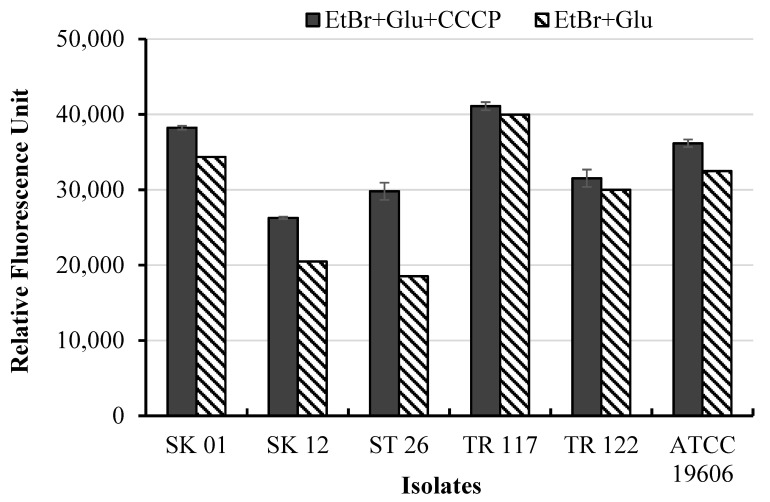
Fluorescence intensity of cells exposed to ethidium bromide in the presence and absence of carbonyl cyanide 3-chlorophenyl hydrazone.

Furthermore, uptake of ethidium bromide, an indicator of AdeABC efflux inhibition, was investigated [32] (Figure 3). Binding of EtBr to double-stranded DNA resulted in a substantial increase in fluorescence for CCCP-treated cells compared with untreated cells. Isolates SK01, SK12, and ST26 showed a significant increase in EtBr uptake after de-energizing with CCCP (*p <* 0.05). The results suggested the likely presence of efflux pumps, as a possible mechanism mediating the resistance of the isolates to fosfomycin.

### 2.7. Synergistic Effects of Fosfomycin-Antibiotics Combination

Several pathogenic bacteria have evolved mechanisms to neutralize or evade the effects of antibiotics. Presently, management of infections caused by *A. baumannii* relies on the use of last-resort antibiotics or administration of multiple antibiotic combinations. Thus, fosfomycin was combined in vitro with 12 conventional antibiotics and evaluated for synergistic effects. The results were interpreted based on the fractional inhibitory concentration index (Table 3).

The antibacterial activities of fosfomycin in combination with carbapenems (imipenem, doripenem, and meropenem) were mainly additive, except the doripenem combination, with FICI vales of 0.31 to 0.50. The fosfomycin and imipenem combination was not synergistic on any isolate, whereas fosfomycin with meropenem showed an FICI range of 0.38 to 0.50 for the isolate SK12. Combinations of fosfomycin with aminoglycosides (gentamicin, amikacin, and tobramycin) presented synergistic effects, with FICI values ranging from 0.25 to 0.5. The results showed that fosfomycin plus gentamicin was synergistic against four isolates, while the fosfomycin plus amikacin combination and the fosfomycin plus tobramycin combination showed synergistic antibacterial effects against two and three isolates, respectively. Similarly, fosfomycin plus glycylcycline combinations (tigecycline or minocycline) displayed synergistic effects against three isolates each, with FICI values ranging from 0.14 to 0.5 and 0.25 to 0.5 for the tigecycline and minocycline combination, respectively. Furthermore, the results revealed the synergistic effects of fosfomycin plus fluoroquinolone (ciprofloxacin or levofloxacin) combinations against two isolates each, and FICI values of 0.31 to 0.5 for the ciprofloxacin combination and 0.25 to 0.5 for the levofloxacin combination. Fosfomycin plus colistin in combination yielded synergistic effects against three isolates with FICI values of 0.25 to 0.5, whereas the combination of fosfomycin and trimethoprim/sulfamethoxazole yielded no synergism.

### 2.8. Time-Kill Kinetics of Combinations of Fosfomycin on A. baumannii

**Figure 4:**
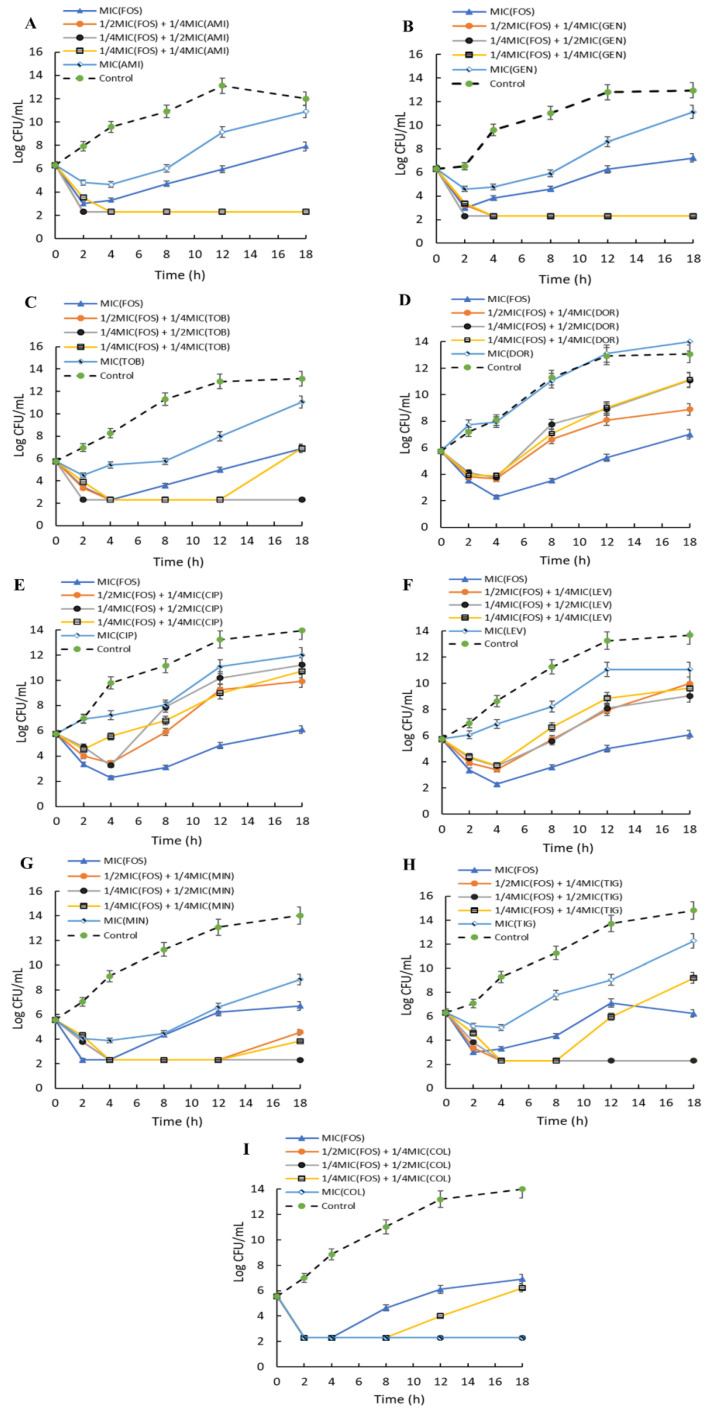
Time-kill curve of fosfomycin combinations against multi-drug-resistant (MDR) clinical isolates of *Acinetobacter baumannii.* (**A**) Fosfomycin and amikacin, (**B**) Fosfomycin and gentamicin, (**C**) Fosfomycin and tobramycin, (**D**) Fosfomycin and doripenem, (**E**) Fosfomycin and ciprofloxacin, (**F**) Fosfomycin and levofloxacin, (**G**) Fosfomycin and minocycline, (**H**) Fosfomycin and tigecycline, and (**I**) Fosfomycin and colistin. The experiments were performed in triplicate and reported as CFU/mL.

The time-dependent killing of fosfomycin in combination with amikacin, gentamicin, tobramycin, doripenem, colistin, levofloxacin, ciprofloxacin, tigecycline, and minocycline was monitored over an 18-h exposure (Figure 4). The results revealed the substantial synergistic effects of fosfomycin in combination with all three aminoglycosides (amikacin, gentamicin, and tobramycin) (Figure 4A–C). At 12 h exposure time, ½ MIC (FOS) + ¼ MIC (AMI), ¼ MIC (FOS) + ½ MIC (AMI), and ¼ MIC (FOS) + ¼ MIC (AMI) demonstrated synergistic bactericidal effects with a ≥3 log reduction in CFU/mL when compared with the MIC of individual antibiotics. Similar results were observed for gentamicin and tobramycin, with synergistic bactericidal effects against the tested isolate. However, ¼ MIC (FOS) + ¼ MIC (TOB) showed regrowth after 12 h of inhibition (Figure 4C). At the concentrations used, the combinations of fosfomycin with either doripenem, levofloxacin, or ciprofloxacin were not synergistic against the *A. baumannii* isolate (TR 122). However, the combination with fosfomycin displayed an additive effect and enhanced the antibacterial activities of doripenem, levofloxacin, and ciprofloxacin with >3 log reduction in CFU/mL when compared with the single antibiotic MIC of doripenem or levofloxacin, and a 1–3 log reduction in CFU/mL for ciprofloxacin. Combinations of fosfomycin and glycylcycline antibiotics (tigecycline and minocycline) demonstrated synergistic bactericidal (>3 log reduction in CFU/mL) effects at ½ MIC (FOS) + ¼ MIC (MIN), ¼ MIC (FOS) + ½ MIC (MIN), and ¼ MIC (FOS) + ¼ MIC (MIN) for minocycline, and ½ MIC (FOS) + ¼ MIC (TIG), ¼ MIC (FOS) + ½ MIC (TIG) for tigecycline at 12 h exposure. In addition, bactericidal effects were observed at the MIC of colistin alone and in combination of fosfomycin with colistin at ½ MIC (FOS) + ¼ MIC (COL), ¼ MIC (FOS) + ½ MIC (COL). At ¼ MIC (FOS) + ¼ MIC of tobramycin, colistin, or tigecycline, the test isolate was inhibited for the first 8 to 12 h before subsequent regrowth at 18 h. 

## 3. Discussion

Antimicrobial drug resistance is ranked as one of the top 10 global health threats facing humanity [33]. Clinical management of hospital-related infections caused by pathogenic Gram-negative *Enterobacterales* and *A. baumannii* uses administration of carbapenems as a last-resort remedy. However, the emergence of carbapenem resistance due to the production of carbapenamase enzymes limits the continued usage of carbapenem antibiotics. Antibacterial testing against carbapenems revealed greater than 90% resistance. Similar results were reported for *A. baumannii* isolates obtained from tertiary hospitals within Thailand [34]. Considering the severity of carbapenem resistance and the dangers it poses, carbapenem-resistant *Enterobacterales* and *A. baumannii* are classified in the critical tier of antimicrobial-resistant pathogens [5].

As treatment options continue to dwindle, the use of combination therapies has become a reliable strategy for the management of drug-resistant pathogens. In addition, antimicrobial combinations subdue the development of resistance by limiting the mutant selection window of single agents [35]. Revitalization of old, abandoned, and somewhat ineffective antibiotics, through arrays of chemical modification, antibiotic hybridizing, or as adjunctive therapies, promises reliable outcomes [36,37]. Although developed nations are restricted by legislations regarding the choice and usage of antibiotics, clinical practitioners in most developing nations are at liberty to administer combinations of antibiotic medications based on experience of previous efficacy.

Fosfomycin, an old phosphonic acid derivative, exhibits enhanced tissue penetration due to its low molecular weight (138 Da) [38] and relatively mild side effects. The in vitro and in vivo antimicrobial effects of fosfomycin monotherapy and in combination have been demonstrated for Gram-positive MDR bacterial isolates including methicillin-resistant *Staphylococcus aureus* [39,40] and Gram-negative *Enterobacterales* [41,42]. However, *A. baumannii* is reported to be intrinsically resistant to fosfomycin monotherapy [43]. The present study investigated the antibacterial effects of fosfomycin alone and the combinatory effects of fosfomycin with regularly used antibiotics as a last-line option for the treatment of infections caused by carbapenem-resistant *A. baumannii*. Fosfomycin alone showed MIC values ranging from 32 to >2048 µg/mL against the 191 isolates. Currently, fosfomycin is listed as a miscellaneous agent for the manage-ment of *A. baumannii*; however, breakpoints have not yet been established. Based on fosfomycin breakpoint values for *Enterobacterales*, the isolates showed a high rate of resistance to fosfomycin, with 57.59% classified as intermediate and 32.98% as resistant. Similar antibacterial effects of fosfomycin against *A. baumannii* have been previously reported [26,31,43]. Several factors, including the presence of efflux pumps [31], resistance-encoded plasmids [44], and mutations in the *ampD* and *anmK* genes, encoding enzymes of the peptidoglycan recycling pathway [43], have been reported to mediate *A. baumannii* resistance to fosfomycin. However, the mechanism of re-sistance is still poorly understood. The MIC values of fosfomycin decreased two- to fourfold, while uptake of ethidium bromide increased in the presence of CCCP. Previous studies have reported reductions in MIC values in the presence of CCCP as a positive indicator of efflux pump-mediated resistance [20,45,46]. Carbonyl cyanide 3-chlorophenyl hydrazone is a known proton motive force and resistance-nodulation-division efflux pump inhibitor that promotes the transport of molecules across the bacteria membrane.

Combinations of fosfomycin with other antibiotics yielded synergistic antibacterial effects against the tested isolates. In particular, combinations of fosfomycin with aminoglycosides demonstrated enhanced bactericidal effects when compared with antibiotics of other classes. This demonstrates the role of a multi-target mechanism in the management of antibiotic-resistant pathogens. Similar findings were reported for combination therapies of fosfomycin and aminoglycosides (amikacin or gentamicin) against MDR bacterial isolates including *Klebsiella pneumoniae*, *Pseudomonas aeruginosa*, and *Escherichia coli* [20,47,48]. Aminoglycosides generally inhibit protein synthesis by binding to the A-site on the 16S ribosomal RNA of the 30S ribosome [49], whereas fosfomycin inhibits the MurA enzyme and UDP-N-acetylglucosamine-enolpyruvyltransferase, involved in peptidoglycan synthesis [50]. In addition, antibiotics of the glycylcycline group (minocycline and tigecycline) and colistin in combination with fosfomycin exhibited synergy against the test isolate. Both the checkerboard techniques and the time-kill assays used to evaluate the in vitro efficacies of the antibiotic combinations showed that fosfomycin with either of amikacin, gentamicin, tobramycin tigecycline, minocycline, or colistin could be used for the management of the carbapenem-resistant *A. baumannii*. The time-kill kinetics further showed additive effects for combinations of fosfomycin and fluoroquinolones (ciprofloxacin or levofloxacin), with less reduction of bacterial count (CFU/mL) compared with fosfomycin monotherapy at MIC. In addition, combinations of fosfomycin with carbapenem (imipenem, meropenem or doripenem) displayed synergistic effects, with two- to fourfold reductions in fosfomycin MICs, as demonstrated by the checkerboard technique. Contrarily, when the doripenem plus fosfomycin combination was assayed with time-kill kinetics, the combination failed to meet the criteria for synergy. Similar inconsistencies between the FICI and time-kill techniques have been reported by previous researchers [51,52]. This observation might be due to the presence of persistent cells that remain viable when the level of antibiotics drops [53]. The addition of carbapenem did not yield effective experimental outcomes, thus co-administration or adjunctive therapy of fosfomycin in *A. baumannii*-infected patients receiving carbapenems might not potentiate favorable clinical outcomes. However, combination therapy might completely kill *A. baumannii* if a longer treatment time is used [53].

## 4. Materials and Methods

### 4.1. Chemicals and Media

All culture media were purchased from Becton Dickinson & Co. Difco (Franklin Lakes, NJ, USA). Colistin sulfate, minocycline hydrochloride, doripenem, and tobramycin were obtained from Sigma-Aldrich, (Saint Louis, MO, USA). Ciprofloxacin, and levofloxacin were purchased from Siam Bheasach Co., Ltd. (Bangkok, Thailand). Tigecycline was purchased from Pfizer Inc. (Philadelphia, PA, USA). Imipenem was obtained from Merck Sharp & Dohme Corp. (Elkton, VA, USA). Meropenem was obtained from M&H manufacturing Co., Ltd. (Samutprakarn, Thailand). Fosfomycin was obtained from Meiji Seikakaisna, Ltd. (Tokyo, Japan).

### 4.2. Bacterial Strains

The study included 193 *A. baumannii* isolates collected from patients admitted to hospitals within Southern Thailand. All isolates were presumptively identified as *Acinetobacter* species using standard biochemical tests as Gram-negative, oxidase-negative, nonmotile, non-fermenting coccobacilli [54], and further identified as *A. baumannii* by matrix-assisted laser desorption ionization-time of flight mass spectrometry (MALDI-TOF-MS). *Acinetobacter baumannii* ATCC 19606 was used as a quality control. All the bacterial cultures were stored in tryptic soy broth (TSB) supplemented with 40% glycerol and kept at −80 °C.

### 4.3. Resistance to Carbapenems

The resistance of the 193 *A. baumannii* isolates to carbapenem was assessed by disc diffusion assay as recommended [55], using a 10-μg disc of imipenem and meropenem. The isolates were cultured to log phase and the cultures were adjusted to 10^6^ CFU/mL in phosphate buffer solution. An aliquot (100 µL) of adjusted culture was evenly spread on Mueller–Hinton agar, and the disc was properly positioned. Plates were incubated at 35 °C for 16 to 18 h, and the zone of inhibition was measured and interpreted.

### 4.4. Screening for Fosfomycin Resistance

The minimum inhibitory concentrations (MICs) of fosfomycin on the 193 isolates was determined by the broth microdilution method in accordance with Clinical and Laboratory Standards Institute (CLSI) guidelines [55]. Briefly, serial two-fold dilutions of antibiotics were prepared in cation-adjusted Mueller–Hinton II broth. Aliquots (100 μL) of the diluted bacterial suspension (1 × 10^6^ CFU/mL) were exposed to 100 μL of varying antibiotic concentrations and incubated at 37 °C for 18 h. MIC was expressed as the lowest concentration of the antibiotic without microbial growth as indicated by the resazurin test. MIC_50_ was defined as the lowest concentration of fosfomycin that inhibited 50% of the isolates, and MIC_90_ as the lowest concentration that inhibited 90% of the isolates.

Additionally, in line with the recommendations for antimicrobial testing of fosfomycin [55], the antibacterial activities of fosfomycin were evaluated on highly resistant isolates with MIC ≥ 512 µg/mL. Mueller-Hinton agar was supplemented with 25 µg/mL of glucose-6-phosphate and varying concentrations of fosfomycin (128–2048 µg/mL). Plates were dried overnight and inoculated with 10^4^ CFU of each isolate. MIC was recorded as the lowest fosfomycin concentration that completely inhibited growth, disregarding a single colony or a faint haze caused by the inoculum. The MICs of fosfomycin in the presence of 12.5 and 25 µg/mL carbonyl cyanide 3-chlorophenyl hydrazone (CCCP) (Sigma-Aldrich, USA) was evaluated.

### 4.5. Antibiogram of Fosfomycin-Resistant Isolates

Fosfomycin-resistant isolates were exposed to 12 conventional antibiotics including carbapenem (imipenem, meropenem, and doripenem), aminoglycosides (amikacin, gentamycin, and tobramycin), glycylcyclines (minocycline and tigecycline), fluroquinolone (ciprofloxacin and levofloxacin), colistin, and trimethoprim/sulfamethoxazole. The minimum inhibitory concentrations of the antibiotics on fosfomycin-resistant isolates were determined using the broth microdilution method as previously detailed.

### 4.6. Ethidium Bromide Uptake Assay

Uptake of ethidium bromide (EtBr) in the presence and absence of CCCP was further investigated as described [31]. In brief, cells were grown to log phase, harvested, and washed thrice with a phosphate buffer solution. Cells were resuspended into PBS and adjusted to 0.3 OD at 600 nm. Afterwards, 1 mL of bacterial suspension was treated with EtBr at 2 µg/mL and incubated for 20 min; 0.4% (*w/v*) of glucose and 25 µg/mL CCCP were added and incubated for 30 min. The cell suspension was then aliquoted into 96-well plates and the fluorescence intensity was measured at excitation and emission of 513 and 600 nm. Cells treated with 0.1% dimethyl sulfoxide without CCCP treatment were used as controls for ethidium accumulation.

### 4.7. Checkerboard Technique

The effects of fosfomycin in combination with 12 other antibiotics (imipenem, meropenem, doripenem, amikacin, gentamycin, tobramycin, minocycline, tigecycline, ciprofloxacin, levofloxacin, colistin, and trimethoprim/sulfamethoxazole) on carbapenem- and fosfomycin-resistant isolates of *A. baumannii* were evaluated using the checkerboard technique. Briefly, 100 μL of diluted bacterial suspension (1 × 10^6^ CFU/mL) was added to wells containing 50 μL of subinhibitory concentrations of fosfomycin and 50 μL of subinhibitory concentrations of one of the 12 other antibiotics. The plates were incubated for 18 h at 37 °C. Inhibitory concentrations were determined as concentrations without growth as indicated by the resazurin test. The antibacterial effects of single antibiotics were tested as a control. The experiment was performed for three independent repeats. The effects of the antimicrobial combination were defined according to the fractional inhibitory concentration index (FICI) as shown in the following equation:

$$\mathrm{FICI}=\frac{\mathrm{MIC}\;\mathrm{of}\;\mathrm{drug}\;A\;\mathrm{in}\;\mathrm{combination}}{\mathrm{MIC}\;\mathrm{of}\;\mathrm{drug}\;A\;\mathrm{alone}}\;+\;\frac{\mathrm{MIC}\;\mathrm{of}\;\mathrm{drug}\;B\;\mathrm{in}\;\mathrm{combination}}{\mathrm{MIC}\;\mathrm{of}\;\mathrm{drug}\;B\;\mathrm{alone}}$$

The FICI results for each combination were interpreted as follows: FICI ≤ 0.5, synergism; 0.5 < FICI < 1, additive; 1 ≤ FICI < 2, indifference; FICI ≥ 2, antagonism. *Escherichia coli* ATCC 25922 was used as a standard control strain for the assays [56].

### 4.8. Time-Kill Assay

Time-kill assays was performed in cation-adjusted Mueller–Hinton broth using the checkerboard assay. Overnight culture of the isolate with the highest MIC of fosfomycin (TR122) was adjusted to ~10^6^ CFU/mL and treated with single antibiotics at MIC and a combination of fosfomycin and other antibiotics at ½ MIC and ¼ MIC. Changes in bacterial population were monitored by plate count at 2, 4, 8, 12, and 18 h, and reported as log reductions in CFU/mL. Untreated cultures were included as a control, and the experiment was performed in triplicate. Antibiotic combination synergism was defined as a 2-log reduction in CFU/mL when compared with the most active single antibiotic, whereas bactericidal activity was defined as a ≥3 log reduction in CFU/mL when compared with the number of viable cells at time zero (0 h) [29].

### 4.9. Ethical Statement

This study was approved by the Institutional Review Board of the Faculty of Medicine, Prince of Songkla University (EC:54-080-14-1-2). The study was conducted at Songklanagarind Hospital, which is an 800-bed university hospital and referral medical center located in southern Thailand. The researchers were granted permission to extract the data from the database with a waiver of consent because of the observational nature of the study. All data were fully anonymized before the researcher accessed and analyzed them. The medical records of adult patients (age ≥ 18 years) seeking medical treatment between February and July 2019 and diagnosed with *A. baumannii* bacteremia were collected between February and December 2019 and used in the study.

## 5. Conclusions

Fosfomycin-based combination therapy might serve as an option for the treatment of MDR *A. baumannii*. Further steps including in vivo efficacy and toxicity in experimental models of infection are required prior to clinical applications.

## Figures and Tables

**Table 1:** Minimum inhibitory concentrations of fosfomycin against carbapenem- and fosfomycin-resistant *Acinetobacter baumannii* isolates evaluated using the agar dilution method and broth microdilution with and without carbonyl cyanide 3-chlorophenyl hydrazine (CCCP).

Isolates	Broth Dilution µg/mL	Agar Dilution µg/mL	+CCCP (25 µg/mL)	+CCCP (12.5 µg/mL)
SK01	512	512	128	128
SK12	512	512	128	128
ST26	>2048	>2048	ND	ND
TR117	512	512	64	128
TR122	1024	512	128	256
ATCC 19606	128	128	128	128

**Table 2:** Minimum inhibitory concentrations of fosfomycin-resistant *A. baumannii* isolates against conventional antibiotics.

Isolates	Antibiotics (µg/mL)											
	Carbapenems			Polymyxin	Aminoglycosides			Folate Inhibitor	Fluoroquinolones		Glycylcycline	
	IMI	MER	DOR	COL	AMI	GEN	TOB	TMS	CIP	LEV	MIN	TIG
SK01	32(R)	32(R)	16(R)	1(S)	4(S)	128(R)	32(R)	>16/80(R)	>16(R)	16(R)	0.5(S)	4(S)
SK12	32(R)	8(R)	8(R)	2(I)	8(S)	256(R)	64(R)	>16/80(R)	2(I)	1(S)	8(I)	8(I)
ST26	>128(R)	>128(R)	>128(R)	2(I)	2(S)	32(R)	8(I)	>16/80(R)	>128(R)	32(R)	16(R)	8(S)
TR117	>128(R)	>128(R)	64(R)	2(I)	1(S)	1(S)	0.5(S)	>16/80(R)	32(R)	4(I)	1(S)	4(S)
TR122	>128(R)	>128(R)	64(R)	2(I)	1(S)	0.5(S)	0.5(S)	>16/80(R)	16(R)	4(I)	0.25(S)	4(S)
ATCC 19606	>128(R)	>128(R)	>128(R)	2(I)	256(R)	4(S)	1(S)	<2/38(S)	> 32(R)	32(R)	0.5(S)	8(I)

R, resistant; S, susceptible; I, intermediate; IMI, Imipenem; MER, Meropenem; DOR, Doripenem; COL, Colistin; AMI, Amikacin; GEN, Gentamicin; TOB, Tobramycin; TMS, Trimethoprim/Sulfamethoxazole; CIP, Ciprofloxacin; LEV, Levofloxacin; MIN, Minocycline; TIG, Tigecycline.

**Table 3:** Combinatory antibacterial activity of subinhibitory concentrations of fosfomycin with conventional antibiotics on carbapenem and fosfomycin-resistant *Acinetobacter baumannii.*

Isolates	Carbapenems					Aminoglycosides					Glycylcyclines					Fluoroquinolones					Polymyxin				
	FOS	µg/mL	FICI	Result	Fold Reduction	FOS	µg/mL	FICI	Result	Fold Reduction	FOS	µg/mL	FICI	Result	Fold Reduction	FOS	µg/mL	FICI	Result	Fold Reduction	FOS	µg/mL	FICI	Result	Fold Reduction
		**IMI**					**GEN**					**TIG**					**CIP**					**COL**			
SK01	256	8	0.75	A	2	256	0.5	0.50	A	2	256	0.5	0.63	A	2	**ND**	**ND**	**ND**	**ND**	ND	256	0.031	0.53	A	2
		4	0.63	A	2		1	0.51	A	2	128	0.5	0.38	S	4							0.062	0.56	A	2
	128	16	0.75	A	4		2	0.52	A	2		1	0.50	S	4						128	0.25	0.50	S	4
	64	16	0.63	A	8		4	0.53	A	2												0.5	0.75	A	4
						128	8	0.31	S	4											64	0.5	0.63	A	8
							16	0.38	S	4															
							32	0.50	S	4															
		**DOR**					**AMI**					**MIN**					**LEV**								
	256	0.25	0.52	A	2	256	0.062	0.52	A	2	256	0.015	0.53	A	2	256	4	0.75	A	2					
		0.5	0.53	A	2	128	0.125	0.28	S	4		0.031	0.56	A	2		8	1	I	2					
		1	0.56	A	2		0.25	0.31	S	4	128	0.031	0.31	S	4										
	128	4	0.50	S	4		0.5	0.38	S	4		0.062	0.37	S	4										
		8	0.75	A	4		1	0.50	S	4		0.125	0.50	S	4										
						64	1	0.38	S	8	64	0.125	0.38	S	4										
						32	1	0.31	S			0.25	0.63	A	8										
		**MER**					**TOB**																		
	256	4	0.63	A	2	256	0.062	0.50	A	2															
	128	16	0.75	A	4	128	2	0.31	S	4															
	64	16	0.63	A	8		4	0.38	S	4															
							8	0.50	S	4															
SK12		**IMI**					**GEN**					**TIG**					**CIP**					**COL**			
	256	4	0.63	A	2	256	64	0.75	A	2	128	4	0.75	A	4	**ND**	**ND**	**ND**	**ND**	ND	128	1	0.75	A	4
		2	0.56	A	2	128	128	0.75	A	4	64	4	0.63	A	8						64	1	0.63	A	8
		1	0.53	A	2		64	0.50	S	4	32	4	0.56	A	16										
	128	16	0.75	A	4																				
		**DOR**					**AMI**					**MIN**					**LEV**								
	256	4	1.00	I	2	64	4	0.63	A	8	256	4	1.00	I	2	256	0.5	1.00	I	2					
		2	0.75	A	2	32	4	0.56	A	16							0.25	0.75	A	2					
	128	4	0.75	A	4											128	0.5	0.75	A	4					
		2	0.50	S	4											64	0.5	0.63	A	8					
	64	4	0.63	A	8											32	0.5	0.56	A	16					
		**MER**					**TOB**																		
	256	0.5	0.56	A	2	256	32	1.00	I	2															
	128	2	0.50	S	4	128	32	0.75	A	4															
		1	0.38	S	4																				
	64	4	0.63	A	8																				
	32	4	0.56	A	16																				
ST26		**IMI**					**GEN**					**TIG**					**CIP**					**COL**			
	**ND**	**ND**	**ND**	**ND**		**ND**	**ND**	**ND**	**ND**	ND	**ND**	**ND**	**ND**	**ND**	ND	**ND**	**ND**	**ND**	**ND**	ND	**ND**	**ND**	**ND**	**ND**	ND
		**DOR**					**AMI**					**MIN**					**LEV**								
	**ND**	**ND**	**ND**	**ND**		**ND**	**ND**	**ND**	**ND**	ND	**ND**	**ND**	**ND**	**ND**	ND	**ND**	**ND**	**ND**	**ND**	ND					
		**MER**					**TOB**																		
	**ND**	**ND**	**ND**	**ND**		**ND**	**ND**	**ND**	**ND**	ND															
TR117		**IMI**					**GEN**					**TIG**					**CIP**					**COL**			
	**ND**	**ND**	**ND**	**ND**	ND	256	0.062	0.56	A	2	256	0.5	0.63	A	2	256	0.5	0.52	A	2	128	0.5	0.50	S	4
							0.031	0.53	A	2		0.25	0.56	A	2		0.25	0.51	A	2	64	1	0.63	A	8
						128	0.25	0.50	S	4	128	1	0.50	S	4	128	8	0.50	S	4					
							0.125	0.38	S	4		0.5	0.38	S	4		4	0.38	S	4					
						64	0.25	0.38	S	8		0.25	0.31	S	4		2	0.31	S	4					
						32	0.25	0.31	S	16	64	1	0.38	S	8	64	8	0.38	S	8					
												0.5	0.25	S	8										
											32	2	0.56	A	16										
												1	0.31	S	16										
		**DOR**					**AMI**					**MIN**					**LEV**								
	**ND**	**ND**	**ND**	**ND**	ND	256	0.062	0.56	A	2	256	0.062	0.56	A	2	256	0.5	0.63	A	2					
												0.031	0.53	A	2	256	0.25	0.56	A	2					
											128	0.25	0.50	S	4	128	1	0.50	S	4					
							**TOB**					0.125	0.38	S	4	64	1	0.38	S	8					
		**MER**				256	0.062	0.62	A	2		0.062	0.31	S	4										
	**ND**	**ND**	**ND**	**ND**	ND	128	0.125	0.50	S	4		0.031	0.28	S	4										
							0.062	0.37	S	4	64	0.25	0.38	S	8										
						64	0.25	0.63	A	8		0.125	0.25	S	8										
							0.125	0.38	S	8	32	0.5	0.56	A	16										
						32	0.25	0.56	A	16		0.25	0.31	S	16										
TR122		**IMI**					**GEN**					**TIG**					**CIP**					**COL**			
	**ND**	**ND**	**ND**	**ND**	ND	512	0.062	0.63	A	2	512	1	0.75	A	2	512	4	0.75	A	2	512	0.5	0.75	A	2
		**DOR**					0.031	0.56	A	2		0.5	0.63	A	2		2	0.63	A	2		0.25	0.63	A	2
	512	4	0.56	A	2	256	0.125	0.50	S	4		0.25	0.56	A	2		1	0.56	A	2		0.125	0.56	A	2
		2	0.53	A	2		0.062	0.38	S	4		0.125	0.53	A	2							0.062	0.53	A	2
	256	16	0.50	S	4	128	0.25	0.63	A	8		0.062	0.52	A	2		**LEV**				256	0.5	0.50	S	4
		8	0.38	S	4		**AMI**				256	1	0.50	S	4	512	1	0.75	A	2		0.25	0.38	S	4
		4	0.31	S	4	512	0.25	0.75	A	2		0.5	0.38	S	4		0.5	0.63	A	2					
	128	32	0.63	A	8		0.125	0.63	A	2		0.25	0.31	S	4		0.25	0.56	A	2					
		**MER**				256	0.25	0.50	S	4		0.125	0.28	S	4		0.125	0.53	A	2					
	**ND**	**ND**	**ND**	**ND**	ND		0.125	0.38	S	4		0.062	0.27	S	4	256	1	0.50	S	4					
						128	0.25	0.38	S	8	128	2	0.63	A	8		0.5	0.38	S	4					
												1	0.38	S	8										
							**TOB**					0.5	0.25	S	8										
						512	0.062	0.62	A	2		**MIN**													
						256	0.125	0.50	S	4	512	0.0312	0.63	A	2										
						128	0.125	0.38	S	8		0.0156	0.56	A	2										
						64	0.25	0.56	A	16	256	0.0625	0.50	S	4										
												0.0312	0.37	S	4										

FOS, fosfomycin; IMI, imipenem; MER, meropenem; DOR, doripenem; COL, colistin; AMI, amikacin; GEN, gentamicin; TOB, tobramycin; TMS, trimethoprim/sulfamethoxazole; CIP, ciprofloxacin; LEV, levofloxacin; MIN, minocycline; TIG, tigecycline; ND, not determined; FICI, fractional inhibitory concentration index; A, additive; S, synergistic; I, indifferent. Bold is used to highlight classes of antibiotics and column headings.

## Data Availability

Data is contained in the article.

## Supplementary Materials

The following are available online at https://www.mdpi.com/1424-8247/14/3/185/s1. Table S1. Demographic and clinical information and outcome of patients. Table S2. Antibacterial effects of carbapenem (imipenem and meropenem) and minimum inhibitory concentrations of fosfomycin on clinical *Acinetobacter baumannii* isolates.
