# Supplementary material for: Evaluation of the Synergistic Antibacterial Effects of Fosfomycin in Combination with Selected Antibiotics against Carbapenem–Resistant Acinetobacter baumannii
Source: Pharmaceuticals (Basel). 2021 Feb 25;14(3):185. doi: 10.3390/ph14030185 (PMC7996625; doi:10.3390/ph14030185)
Supplement: Supplementary file 1 [file pharmaceuticals-14-00185-s001.zip › pharmaceuticals-1115578-supplementary.pdf]

Table S1. Demographic and clinical information and outcome of patients

Isolates	Hospital	Site	Sample type	Sex	APACHE II	Admission	Initial ward	Service	Discharge status	Comorbidities
SK001	PSU	Sputum	CS	M	17	Emergency	ICU	Medicine	Survived	DM, HTN, DLD
SK002	Songkhla	Sputum	C	M	17	Elective	General	Medicine	Survived	
SK003	Hatyai	NP	C	M	13	Emergency	ICU	Surgery	Survived	
SK004	Hatyai	Sputum	C	M	12	Emergency	ICU	Medicine	Survived	DM, CVA
SK005	PSU	NG	C	F	21	Emergency	General	Medicine	Dead	DM
SK006	PSU	Sputum	C	F	25	Emergency	ICU	Surgery	Survived	DM
SK009	Hatyai	Blood	CS	F	22	Elective	General	Surgery	Dead	
SK010	Hatyai	Sputum	C	M	23	Emergency	ICU	Surgery	Dead	DM
SK011	PSU	Rectum	C	M	21	Emergency	ICU	Surgery	Dead	DM
SK012	PSU	Sputum	C	F	20	Emergency	ICU	Surgery	Survived	
SK013	PSU	Sputum	C	M	18	Emergency	ICU	Surgery	Survived	HTN, CKD, CAD, COPD
SK014	Hatyai	Skin	C	M	13	Emergency	ICU	Surgery	Dead	
SK015	PSU	Sputum	C	M	15	Emergency	General	Medicine	Dead	DM, DLD
SK016	PSU	Sputum	CS	F	17	Emergency	General	Medicine	Survived	DM
SK022	Songkhla	Sputum	C	M	17	Emergency	General	Medicine	Survived	HIV
SK023	Songkhla	Sputum	C	M	15	Emergency	General	Medicine	Dead	DM
SK024	PSU	Skin	C	M	15	Emergency	ICU	Surgery	Survived	DM
SK025	Hatyai	NP	C	M	17	Emergency	ICU	Medicine	Survived	DM
SK028	Hatyai	NG	C	F	20	Emergency	General	Medicine	Dead	DM, CKD
SK031	Songkhla	Sputum	C	M	23	Emergency	ICU	Medicine	Survived	HTN, COPD
SK032	PSU	Blood	CS	F	27	Emergency	ICU	Surgery	Survived	CAD
SK033	PSU	Urine	CS	F	21	Emergency	ICU	Medicine	Survived	DM, CVA
SK034	Songkhla	Sputum	C	F	20	Emergency	ICU	Medicine	Dead	DM
SK035	Songkhla	Sputum	C	F	18	Elective	General	Medicine	Dead	DM
SK036	Hatyai	NG	C	M	17	Emergency	ICU	Medicine	Dead	
SK037	Songkhla	Sputum	C	M	17	Emergency	ICU	Medicine	Survived	
SK038	Songkhla	Sputum	C	M	23	Elective	General	Medicine	Survived	DM
SK039	Hatyai	Skin	C	M	21	Emergency	ICU	Medicine	Survived	DM, HTN, CKD, CAD
SK040	Hatyai	Sputum	CS	F	22	Emergency	ICU	Medicine	Dead	DM, DLD, COPD
SK041	Hatyai	Sputum	C	M	23	Emergency	ICU	Surgery	Dead	
SK042	Songkhla	NP	C	M	21	Emergency	ICU	Medicine	Survived	DM
SK043	Songkhla	Sputum	C	M	20	Emergency	General	Medicine	Survived	
SK044	Hatyai	Sputum	C	F	15	Emergency	ICU	Medicine	Survived	
SK045	Hatyai	NG	C	M	15	Emergency	ICU	Surgery	Dead	DM
SK049	PSU	Sputum	C	M	17	Emergency	ICU	Surgery	Survived	DM
SK050	PSU	Sputum	C	M	10	Elective	General	Surgery	Survived	DM, HTN, CKD, CVA,
SK052	PSU	Skin	C	M	8	Emergency	ICU	Surgery	Dead	DLD
SK053	PSU	Sputum	CS	M	17	Emergency	ICU	Surgery	Survived	HIV
SK054	PSU	Sputum	C	M	12	Emergency	ICU	Medicine	Dead	COPD
SK055	Hatyai	Sputum	C	F	17	Emergency	General	Medicine	Dead	DM
SK056	Hatyai	Skin	C	M	15	Emergency	ICU	Surgery	Dead	DM, CAD
SK057	Songkhla	Sputum	C	M	15	Emergency	ICU	Medicine	Dead	DM
SK058	PSU	Sputum	C	M	21	Emergency	ICU	Medicine	Survived	
SK059	PSU	NP	C	F	20	Elective	General	Surgery	Survived	HTN
SK060	Songkhla	Sputum	C	F	22	Emergency	ICU	Surgery	Survived	DM
SK061	Hatyai	Wound	CS	F	23	Emergency	ICU	Surgery	Dead	DM, DLD, CKD, CVA
SK062	Hatyai	Sputum	CS	F	21	Emergency	General	Surgery	Survived	DM, COPD
SK063	PSU	Sputum	C	M	20	Emergency	ICU	Medicine	Dead	
SK064	PSU	Sputum	CS	M	21	Elective	general	Medicine	Dead	
SK065	Hatyai	Sputum	C	M	18	Emergency	ICU	Medicine	Survived	
SK066	Songkhla	Skin	C	M	23	Emergency	ICU	Surgery	Survived	DM
SK067	Hatyai	Sputum	C	F	27	Emergency	General	Surgery	Survived	DLD, CKD
SK068	PSU	Blood	CS	M	21	Emergency	ICU	Surgery	Dead	HTN, HIV
SK069	PSU	Sputum	CS	M	20	Emergency	ICU	Surgery	Survived	DM, CVA, CAD
SK070	Songkhla	Sputum	C	F	18	Elective	General	Medicine	Survived	
SK075	PSU	Sputum	CS	M	17	Emergency	ICU	Medicine	Survived	COPD
SK077	Hatyai	Sputum	C	M	17	Emergency	ICU	Medicine	Dead	DM
SK078	Songkhla	Sputum	CS	F	23	Emergency	ICU	Surgery	Dead	
SK079	Hatyai	Sputum	C	M	21	Emergency	ICU	Medicine	Survived	

ST002	Satoon	Sputum	C	M	23	Emergency	ICU	Medicine	Survived	DM
ST004	Satoon	Blood	CS	M	21	Elective	General	Medicine	Survived	DLD
ST005	Pattalung	Sputum	CS	F	20	Emergency	ICU	Medicine	Dead	HTN
ST006	Satoon	Sputum	C	M	21	Emergency	ICU	Surgery	Survived	CKD
ST009	Satoon	Sputum	CS	M	13	Emergency	ICU	Medicine	Survived	DM, CAD
ST010	Pattalung	Blood	CS	M	25	Emergency	General	Medicine	Survived	
ST011	Pattalung	Sputum	CS	M	13	Emergency	General	Medicine	Dead	DLD, COPD, HIV
ST012	Pattalung	Sputum	C	F	21	Emergency	ICU	Medicine	Survived	DM
ST013	Satoon	Skin	C	F	22	Emergency	ICU	Surgery	Survived	
ST016	Satoon	Sputum	C	F	18	Emergency	ICU	Surgery	Survived	
ST017	Satoon	Sputum	CS	M	17	Elective	General	Surgery	Dead	DM
ST018	Pattalung	Sputum	CS	M	18	Emergency	ICU	Surgery	Survived	
ST021	Satoon	Urine	CS	M	21	Emergency	ICU	Medicine	Survived	HTN, DLD, CAD
ST023	Satoon	Sputum	C	M	27	Emergency	General	Medicine	Survived	DM, CKD
ST024	Pattalung	NP	C	F	23	Emergency	ICU	Medicine	Dead	DLD, CKD
ST026	Satoon	Sputum	CS	M	22	Emergency	ICU	Surgery	Survived	
ST027	Satoon	Wound	CS	M	20	Elective	General	Medicine	Survived	COPD
TR0001	Trang	Sputum	C	F	21	Emergency	General	Surgery	Survived	DM, CVA
TR0005	Trang	Sputum	CS	M	18	Emergency	General	Medicine	Survived	
TR0006	Pattani	Sputum	C	M	17	Emergency	ICU	Medicine	Dead	HIV
TR0007	Trang	NG	C	M	15	Emergency	ICU	Medicine	Survived	
TR0009	Yala	Sputum	CS	F	18	Emergency	ICU	Surgery	Survived	DM
TR0010	Pattani	Sputum	C	F	18	Emergency	ICU	Medicine	Survived	HTN, DLD, CKD
TR0011	Trang	Sputum	CS	F	14	Emergency	ICU	Medicine	Dead	
TR0013	Yala	Blood	CS	F	12	Emergency	ICU	Medicine	Survived	HTN
TR0014	Trang	Sputum	CS	M	21	Emergency	General	Surgery	Dead	DM, HTN, DLD, CAD
TR0015	Pattani	Sputum	C	M	25	Elective	General	Medicine	Dead	COPD
TR0017	Trang	Wound	CS	M	22	Emergency	General	Medicine	Survived	
TR0019	Yala	Sputum	C	F	24	Emergency	General	Medicine	Survived	HTN
TR0020	Yala	NP	C	M	21	Emergency	ICU	Surgery	Survived	DM, HTN, DLD, CKD, HIV
TR0021	Narathiwas	Sputum	C	F	20	Emergency	ICU	Medicine	Survived	HTN, CVA
TR0022	Trang	Sputum	CS	F	18	Emergency	ICU	Medicine	Dead	
TR0023	Pattani	Sputum	CS	M	14	Emergency	ICU	Medicine	Survived	DM, CAD
TR0025	Trang	Sputum	C	M	15	Elective	General	Surgery	Dead	
TR0026	Trang	Blood	CS	M	18	Emergency	ICU	Surgery	Dead	HTN
TR0027	Yala	Sputum	C	F	18	Emergency	ICU	Surgery	Dead	
TR0028	Narathiwas	Sputum	CS	M	15	Emergency	ICU	Medicine	Dead	DM, DLD, COPD
TR0029	Trang	Sputum	C	M	15	Emergency	ICU	Surgery	Dead	HTN
TR0030	Pattani	Sputum	CS	M	18	Emergency	ICU	Surgery	Dead	
TR0031	Trang	Sputum	CS	F	20	Emergency	ICU	Surgery	Dead	
TR0032	Yala	Sputum	C	M	24	Emergency	ICU	Medicine	Survived	DM, CKD, CAD
TR0033	Trang	Sputum	CS	M	28	Emergency	ICU	Medicine	Survived	HTN, HIV
TR0034	Yala	Urine	CS	M	21	Emergency	General	Medicine	Survived	
TR0035	Trang	Sputum	CS	M	20	Elective	General	Medicine	Dead	
TR0036	Pattani	Sputum	C	F	18	Emergency	General	Surgery	Survived	DM
TR0037	Trang	Sputum	C	M	18	Emergency	ICU	Surgery	Survived	HTN, DLD, CKD
TR0038	Trang	Skin	C	M	18	Emergency	ICU	Surgery	Dead	HTN
TR0039	Yala	Sputum	CS	M	24	Emergency	ICU	Medicine	Survived	DM, COPD
TR0040	Yala	Blood	CS	F	21	Emergency	ICU	Surgery	Survived	
TR0041	Trang	Sputum	CS	M	22	Emergency	ICU	Surgery	Dead	
TR0042	Narathiwas	Sputum	C	M	24	Elective	General	Medicine	Survived	DM, HTN, CAD
TR0043	Trang	Sputum	CS	M	21	Emergency	ICU	Surgery	Survived	HTN, CVA
TR0044	Trang	Blood	CS	M	20	Emergency	ICU	Surgery	Survived	
TR0045	Pattani	Sputum	CS	M	15	Emergency	ICU	Medicine	Dead	
TR0046	Trang	Sputum	CS	F	15	Emergency	ICU	Medicine	Dead	HIV
TR0047	Yala	Wound	CS	M	18	Emergency	ICU	Surgery	Survived	DM, HTN, DLD
TR0048	Trang	Sputum	CS	M	10	Emergency	ICU	Medicine	Dead	
TR0049	Narathiwas	Sputum	C	M	8	Emergency	General	Medicine	Survived	CKD, COPD
TR0050	Narathiwas	NP	C	F	18	Elective	General	Medicine	Survived	
TR0051	Trang	Sputum	C	M	12	Emergency	ICU	Medicine	Dead	DM, HTN
TR0052	Yala	Wound	CS	M	18	Emergency	ICU	Surgery	Dead	
TR0053	Trang	Sputum	CS	M	15	Emergency	ICU	Medicine	Survived	DM, HTN, CAD
TR0054	Trang	Sputum	CS	M	15	Emergency	ICU	Medicine	Survived	DM

TR0055	Pattani	Sputum	C	F	21	Emergency	General	Medicine	Survived	DM, HTN, DLD
TR0056	Trang	NP	C	M	20	Emergency	ICU	Surgery	Dead	
TR0057	Narathiwas	NP	C	M	22	Emergency	ICU	Medicine	Survived	
TR0058	Trang	Sputum	C	M	24	Emergency	ICU	Medicine	Survived	CKD
TR0059	Yala	Blood	CS	M	21	Elective	General	Medicine	Dead	HTN, DLD, COPD
TR0060	Trang	Sputum	CS	F	20	Emergency	ICU	Surgery	Survived	DM
TR0061	Yala	Sputum	C	M	21	Emergency	ICU	Surgery	Survived	HTN
TR0062	Trang	Skin	C	M	18	Emergency	ICU	Medicine	Dead	HTN
TR0063	Narathiwas	Sputum	CS	M	24	Emergency	ICU	Medicine	Survived	CAD
TR0064	Trang	Urine	CS	F	28	Emergency	ICU	Medicine	Survived	DM
TR0065	Trang	Sputum	C	F	21	Emergency	General	Medicine	Dead	CVA
TR0067	Pattani	Sputum	CS	M	20	Emergency	ICU	Surgery	Survived	HTN, DLD
TR0068	Trang	Sputum	C	M	18	Emergency	ICU	Medicine	Survived	
TR0069	Narathiwas	Sputum	CS	M	18	Emergency	ICU	Medicine	Survived	CKD
TR0070	Yala	Wound	CS	F	18	Elective	General	Medicine	Survived	DM, HTN, HIV
TR0071	Trang	Sputum	CS	M	24	Emergency	ICU	Surgery	Dead	HTN
TR0073	Trang	Sputum	CS	M	21	Emergency	ICU	Surgery	Dead	HTN, DLD, CAD
TR0074	Yala	Sputum	C	M	24	Emergency	ICU	Medicine	Dead	
TR0082	Trang	Wound	CS	M	21	Emergency	ICU	Surgery	Dead	CAD
TR0084	Pattani	Sputum	CS	F	20	Emergency	ICU	Medicine	Dead	
TR0085	Pattani	Sputum	C	M	21	Emergency	ICU	Surgery	Survived	DM
TR0086	Yala	Sputum	C	M	14	Emergency	ICU	Surgery	Survived	
TR0087	Pattani	Sputum	CS	M	25	Emergency	ICU	Surgery	Survived	CKD, CVA
TR0088	Trang	Skin	C	F	14	Emergency	ICU	Medicine	Dead	
TR0089	Trang	NP	C	F	21	Emergency	ICU	Medicine	Survived	
TR0090	Yala	Sputum	C	F	22	Elective	General	Medicine	Survived	
TR0091	Yala	NG	C	F	18	Emergency	ICU	Surgery	Survived	
TR0092	Trang	Blood	CS	F	18	Emergency	ICU	Surgery	Dead	DM
TR0093	Narathiwas	Sputum	C	F	18	Emergency	ICU	Surgery	Survived	
TR0094	Trang	Sputum	CS	F	21	Emergency	ICU	Surgery	Survived	
TR0095	Pattani	Sputum	C	M	28	Emergency	ICU	Medicine	Survived	DM
TR0096	Pattani	Sputum	CS	M	24	Emergency	ICU	Medicine	Dead	HTN
TR0097	Yala	Sputum	C	F	22	Emergency	ICU	Surgery	Survived	
TR0098	Pattani	Sputum	C	M	20	Emergency	ICU	Medicine	Survived	CKD, COPD
TR099	Pattani	Blood	CS	M	21	Emergency	ICU	Medicine	Survived	DM
TR100	Yala	Sputum	C	F	18	Elective	General	Surgery	Dead	DLD
TR0101	Pattani	Sputum	C	M	18	Emergency	ICU	Medicine	Dead	
TR0102	Trang	Sputum	C	M	15	Emergency	ICU	Medicine	Dead	
TR0103	Trang	Sputum	CS	M	17	Emergency	ICU	Medicine	Dead	DLD, CAD
TR0104	Yala	Sputum	C	M	17	Emergency	ICU	Medicine	Survived	DM, HIV
TR0105	Trang	Sputum	CS	F	13	Emergency	ICU	Surgery	Survived	
TR0106	Trang	Urine	CS	M	12	Emergency	ICU	Medicine	Survived	HTN
TR0107	Pattani	Sputum	C	M	21	Emergency	ICU	Medicine	Survived	CKD
TR0108	Trang	Skin	C	M	25	Emergency	ICU	Surgery	Dead	DLD
TR0109	Yala	Sputum	C	F	22	Elective	General	Medicine	Dead	
TR0110	Trang	Sputum	CS	M	23	Emergency	ICU	Medicine	Survived	DLD
TR0111	Trang	Skin	C	M	21	Emergency	ICU	Medicine	Dead	DM, CVA
TR0112	Yala	NP	C	M	20	Emergency	ICU	Medicine	Survived	
TR0113	Trang	Sputum	C	M	18	Emergency	ICU	Surgery	Survived	
TR0114	Trang	Sputum	CS	M	13	Emergency	ICU	Medicine	Survived	
TR0115	Pattani	Sputum	C	F	15	Emergency	ICU	Medicine	Dead	DM, CKD, CAD
TR0116	Trang	NP	C	F	17	Emergency	ICU	Medicine	Survived	DM, HTN
TR0117	Narathiwas	Sputum	C	F	17	Emergency	General	Medicine	Dead	DLD
TR0118	Trang	Sputum	CS	F	15	Elective	General	Surgery	Survived	
TR0119	Yala	Sputum	C	M	15	Emergency	General	Surgery	Survived	
TR0120	Trang	NP	C	M	17	Emergency	ICU	Surgery	Dead	CAD
TR0121	Trang	NG	C	F	20	Emergency	ICU	Medicine	Dead	
TR0122	Trang	Blood	CS	M	23	Emergency	ICU	Medicine	Survived	DM
TR0123	Yala	Skin	C	F	27	Emergency	ICU	Medicine	Survived	CKD
TR0124	Trang	NG	C	F	21	Emergency	ICU	Medicine	Survived	
TR0125	Pattani	NP	C	M	20	Emergency	General	Surgery	Dead	
TR0126	Trang	Sputum	CS	M	18	Emergency	ICU	Surgery	Survived	HTN, COPD
TR0127	Yala	Sputum	C	M	17	Emergency	ICU	Medicine	Survived	DLD

TR0128	Trang	Skin	C	F	17	Emergency	ICU	Surgery	Survived	
TR0129	Pattani	Sputum	C	M	23	Elective	General	Medicine	Survived	DM
TR0130	Narathiwas	Sputum	CS	F	21	Emergency	ICU	Medicine	Survived	
TR0131	Pattani	Sputum	C	M	22	Emergency	ICU	Surgery	Dead	CVA
TR0132	Trang	NG	C	M	23	Emergency	ICU	Medicine	Dead	CKD, CAD
TR0133	Yala	NP	C	F	21	Emergency	ICU	Medicine	Survived	
TR0134	Trang	Sputum	CS	F	20	Emergency	ICU	Surgery	Dead	DM
TR0135	Narathiwas	Sputum	C	M	15	Emergency	ICU	Surgery	Survived	HTN

Site: NG; nasogastric tube, NP; nasopharyngeal swab, Sample type: CS; clinical specimen, C; colonization,

Comorbidities: DM; diabetes mellitus, HTN; essential blood hypertension, DLD; dyslipidaemia, CKD; chronic kidney disease, CVA; cerebrovascular disease, CAD; coronary heart disease, COPD; chronic obstructive pulmonary disease, HIV; human immunodeficiency viral infection, ICU; intensive care unit.

Table S2. Antibacterial effects of carbapenem (imipenem and meropenem) and minimum inhibitory concentrations of fosfomycin on clinical *Acinetobacter baumannii* isolates

S/N	Isolates code	Zone of inhibition (mm)		MIC Fosfomycin (µg/mL)	
		Imipenem	Meropenem		
1	SK001	R	R	512	512
2	SK002	R	R	256	256
3	SK003	S (27.60)	I (17.80)	128	128
4	SK004	S (27.60)	S (18.70)	128	128
5	SK005	R	R	128	128
6	SK006	R	R	256	256
7	SK009	R	R	256	256
8	SK010	R	R	256	256
9	SK011	R	R	256	256
10	SK012	I (20.50)	R	512	512
11	SK013	R	R	256	256
12	SK014	R	R	256	256
13	SK015	R	R	128	128
14	SK016	R	R	256	256
15	SK022	R	R	256	256
16	SK023	S (27.85)	S (30.20)	64	64
17	SK024	R	R	128	128
18	SK025	R	R	128	128
19	SK028	R	R	256	256
20	SK031	R	R	128	128
21	SK032	R	R	256	256
22	SK033	R	R	128	128
23	SK034	R	R	128	128
24	SK035	R	R	256	256
25	SK036	S (26.85)	R	256	256
26	SK037	S (30.25)	R	256	128
27	SK038	R	R	256	256
28	SK039	R	R	256	128
29	SK040	R	R	256	256
30	SK041	R	R	256	256
31	SK042	R	R	256	256
32	SK043	R	R	64	64
33	SK044	R	R	256	256
34	SK045	R	R	128	128
35	SK049	R	R	256	256
36	SK050	R	R	256	256
37	SK052	R	R	128	128
38	SK053	R	R	256	256
39	SK054	R	R	256	256

40	SK055	R	R	256	256
41	SK056	R	R	256	256
42	SK057	R	R	256	256
43	SK058	R	R	256	128
44	SK059	R	R	256	256
45	SK060	R	R	256	256
46	SK061	R	R	256	256
47	SK062	R	R	256	256
48	SK063	R	R	256	256
49	SK064	R	R	256	128
50	SK065	R	R	256	256
51	SK066	R	R	256	128
52	SK067	R	R	256	256
53	SK068	R	R	256	256
54	SK069	R	R	256	256
55	SK070	R	R	256	256
56	SK075	R	R	256	256
57	SK077	R	R	128	128
58	SK078	R	R	256	256
59	SK079	R	R	128	128
60	ST002	R	R	128	128
61	ST004	R	R	64	64
62	ST005	R	R	128	128
63	ST006	S (37.25)	S (46.35)	32	32
64	ST009	R	R	128	128
65	ST010	R	R	64	128
66	ST011	R	R	256	256
67	ST012	R	R	256	128
68	ST013	R	R	128	128
69	ST016	R	R	128	128
70	ST017	R	R	128	128
71	ST018	R	R	128	128
72	ST021	R	R	128	128
73	ST023	S (22.75)	S (29.10)	2048	2048
74	ST024	R	R	128	128
75	ST026	R	R	>2048	>2048
76	ST027	R	R	128	128
77	TR0001	S (26.50)	S (20.00)	64	64
78	TR0005	R	R	256	256
79	TR0006	R	R	256	256
80	TR0007	R	R	128	128
81	TR0009	R	R	128	128
82	TR0010	R	R	128	128
83	TR0011	R	R	128	128
84	TR0013	R (11.60)	R (11.60)	128	128
85	TR0014	R	R	128	128

86	TR0015	R	R	256	256
87	TR0017	R	R	256	256
88	TR0019	R	R	128	128
89	TR0020	R	R	128	128
90	TR0021	R	R	128	128
91	TR0022	R	R	64	64
92	TR0023	R	R	128	128
93	TR0025	R	R	64	64
94	TR0026	R	R	128	128
95	TR0027	R	R	128	128
96	TR0028	R	R	256	256
97	TR0029	R	R	256	256
98	TR0030	S (25.80)	I (17.00)	ND	ND
99	TR0031	S (25.50)	I (16.45)	ND	ND
100	TR0032	R	R	128	128
101	TR0033	R	R	64	64
102	TR0034	R	R	128	128
103	TR0035	R	R	256	256
104	TR0036	R	R	128	128
105	TR0037	R	R	128	128
106	TR0038	R	R	128	128
107	TR0039	R	R	128	128
108	TR0040	R	R	128	128
109	TR0041	R	R	128	128
110	TR0042	R	R	64	64
111	TR0043	R	R	128	128
112	TR0044	R	R	128	128
113	TR0045	R	R	128	128
114	TR0046	R	R	128	128
115	TR0047	R	R	128	128
116	TR0048	R	R	128	128
117	TR0049	R	R	64	64
118	TR0050	R	R	128	128
119	TR0051	R	S (21.00)	64	64
120	TR0052	R	R	64	64
121	TR0053	R	R	128	128
122	TR0054	R	R	128	128
123	TR0055	R	R	128	128
124	TR0056	R	R	128	128
125	TR0057	R	R	128	128
126	TR0058	R	R	256	256
127	TR0059	R	R	128	128
128	TR0060	R	R	128	128
129	TR0061	R	R	128	128
130	TR0062	R	R	256	256
131	TR0063	R	R	128	128

132	TR0064	R	R	64	64
133	TR0065	R	R	128	128
134	TR0067	R	R	64	64
135	TR0068	R	R	128	128
136	TR0069	R	R	128	128
137	TR0070	R	R	128	128
138	TR0071	R	R	64	64
139	TR0073	R	R	128	128
140	TR0074	R	R	128	128
141	TR0082	R	R	128	128
142	TR0084	R	R	128	128
143	TR0085	R	R	64	64
144	TR0086	R	R	128	128
145	TR0087	R	R	128	128
146	TR0088	R	R	128	128
147	TR0089	R	R	128	128
148	TR0090	R	R	128	128
149	TR0091	R	R	128	256
150	TR0092	R	R	128	128
151	TR0093	R	R	256	256
152	TR0094	R	R	128	128
153	TR0095	R	R	128	128
154	TR0096	S (24.65)	S (22.30)	512	512
155	TR0097	R	R	128	128
156	TR0098	R	R	128	128
157	TR099	R	R	128	128
158	TR100	R	R	128	128
159	TR0101	R	R	128	128
160	TR0102	R	R	128	128
161	TR0103	S (24.25)	S (28.55)	128	128
162	TR0104	R	R	256	256
163	TR0105	R	R	256	256
164	TR0106	R	R	128	128
165	TR0107	R	R	128	128
166	TR0108	R	R	64	64
167	TR0109	R	R	128	128
168	TR0110	R	R	128	128
169	TR0111	R	R	64	64
170	TR0112	R	R	128	128
171	TR0113	R	R	128	128
172	TR0114	R	R	128	128
173	TR0115	R	R	128	256
174	TR0116	R	R	128	256
175	TR0117	R	R	512	512
176	TR0118	R	R	128	128
177	TR0119	R	R	128	128

178	TR0120	R	R	256	256
179	TR0121	R	R	256	128
180	TR0122	R	R	1024	1024
181	TR0123	R	R	128	128
182	TR0124	R	R	128	128
183	TR0125	R	R	128	128
184	TR0126	R	R	128	128
185	TR0127	R	R	128	128
186	TR0128	R	R	256	256
187	TR0129	R (11.10)	R (10.40)	256	256
188	TR0130	R	R	128	128
189	TR0131	R	R	128	128
190	TR0132	R	R	128	128
191	TR0133	R	R	128	128
192	TR0134	R	R	128	128
193	TR0135	R	R	128	128
